# Image-based phenotyping for non-destructive screening of different salinity tolerance traits in rice

**DOI:** 10.1186/s12284-014-0016-3

**Published:** 2014-08-14

**Authors:** Aris Hairmansis, Bettina Berger, Mark Tester, Stuart John Roy

**Affiliations:** Australian Centre for Plant Functional Genomics and the School of Agriculture Food and Wine, Waite Campus, University of Adelaide, PMB1 Glen Osmond, Adelaide, 5064 SA Australia; The Plant Accelerator, Australian Plant Phenomics Facility, School of Agriculture Food and Wine, Waite Campus, University of Adelaide, PMB1 Glen Osmond, Adelaide, 5064 SA Australia; Center for Desert Agriculture, Division of Biological and Environmental Sciences and Engineering, King Abdullah University of Science and Technology, Thuwal, 23955-6900 Kingdom of Saudi Arabia

**Keywords:** Rice (Oryza sativa L.), Salinity tolerance, Phenotyping, Image analysis, Growth, Senescence

## Abstract

**Background:**

Soil salinity is an abiotic stress wide spread in rice producing areas, limiting both plant growth and yield. The development of salt-tolerant rice requires efficient and high-throughput screening techniques to identify promising lines for salt affected areas. Advances made in image-based phenotyping techniques provide an opportunity to use non-destructive imaging to screen for salinity tolerance traits in a wide range of germplasm in a reliable, quantitative and efficient way. However, the application of image-based phenotyping in the development of salt-tolerant rice remains limited.

**Results:**

A non-destructive image-based phenotyping protocol to assess salinity tolerance traits of two rice cultivars (IR64 and Fatmawati) has been established in this study. The response of rice to different levels of salt stress was quantified over time based on total shoot area and senescent shoot area, calculated from visible red-green-blue (RGB) and fluorescence images. The response of rice to salt stress (50, 75 and 100 mM NaCl) could be clearly distinguished from the control as indicated by the reduced increase of shoot area. The salt concentrations used had only a small effect on the growth of rice during the initial phase of stress, the shoot Na^+^ accumulation independent phase termed the ‘osmotic stress’ phase. However, after 20 d of treatment, the shoot area of salt stressed plants was reduced compared with non-stressed plants. This was accompanied by a significant increase in the concentration of Na^+^ in the shoot. Variation in the senescent area of the cultivars IR64 and Fatmawati in response to a high concentration of Na^+^ in the shoot indicates variation in tissue tolerance mechanisms between the cultivars.

**Conclusions:**

Image analysis has the potential to be used for high-throughput screening procedures in the development of salt-tolerant rice. The ability of image analysis to discriminate between the different aspects of salt stress (shoot ion-independent stress and shoot ion dependent stress) makes it a useful tool for genetic and physiological studies to elucidate processes that contribute to salinity tolerance in rice. The technique has the potential for identifying the genetic basis of these mechanisms and assisting in pyramiding different tolerance mechanisms into breeding lines.

**Electronic supplementary material:**

The online version of this article (doi:10.1186/s12284-014-0016-3) contains supplementary material, which is available to authorized users.

## Background

Salinity is a major abiotic stress that threatens the sustainability of global rice production. Rice yield can be reduced significantly by the addition of as little as 50 mM NaCl (Yeo and Flowers [1986]), making it one of the crop species most susceptible to salt stress (Grattan et al. [2002]; Munns and Tester [2008]). It has been estimated that about 48 million ha of potentially useful agricultural land is unusable for growing rice in Southern Asia and South East Asia due to saline soils (Ponnamperuma and Bandyopadhya [1980]; Vinod et al. [2013]). The cultivation of salt-tolerant rice is important to maintain the sustainability of rice production in such areas. However, progress with breeding programmes to develop salt-tolerant rice has been slow (Gregorio et al. [2002]; Flowers [2004]; Yamaguchi and Blumwald [2005]). One of the limiting factors in the breeding of salt tolerant rice is the availability of efficient and reliable screening techniques to select tolerant plants (Gregorio et al. [2002]).

A number of screening methods for different morpho-physiological traits have been used to measure salinity tolerance in rice, including shoot weight (Yeo et al. [1990]; Aslam et al. [1993]), shoot Na^+^ concentration, the ratio of shoot Na^+^/K^+^ (Yeo et al. [1988]; Gregorio and Senadhira [1993]; Asch et al. [2000]), leaf injury and survival rate (Yeo et al. [1990]; Gregorio et al. [1997]), leaf area (Akita and Cabuslay [1990]; Zeng et al. [2003]) and bypass flow in the root (Faiyue et al. [2012]). Of these traits, shoot weight was shown to be closely related to overall plant performance (Yeo et al. [1990]) and to the performance of the plant in the field (Aslam et al. [1993]). However, most protocols that measure plant biomass are destructive, thus making it difficult to measure dynamic responses in plant growth in response to salt application and to collect seed from the individuals being measured. Recent developments in image-based phenotyping have enabled the non-destructive assessment of plant responses to salinity over time and allows determination of shoot biomass measurements without having to harvest the whole plant (Rajendran et al. [2009]; Furbank and Tester [2011]; Berger et al. [2012a]; Jansen et al. [2014]).

When plants are exposed to salt, their growth immediately slows due to the shoot ion independent stress, the so-called osmotic component of salt stress, and plants produce fewer tillers (Munns and Tester [2008]; Rajendran et al. [2009]; Horie et al. [2012]). Over time, Na^+^ and Cl^-^ will accumulate to toxic concentrations in the shoot, resulting in premature leaf senescence and death – the ionic component of salt stress (Tester and Davenport [2003]; Munns and Tester [2008]; Munns [2010]; Horie et al. [2012]). Importantly, image-based phenotyping can differentiate between the effects of the osmotic and ionic components of salt stress in growing plants. This can be done by measuring growth responses immediately after salt application, before the accumulation of toxic concentrations of ions in the shoot. This allows for at least some dissection of salinity tolerance mechanisms (Rajendran et al. [2009]; Sirault et al. [2009]).

Several studies have used image based phenotyping to measure salinity tolerance in crops, in particular wheat and barley (Rajendran et al. [2009]; Sirault et al. [2009]; Harris et al. [2010]), where digital colour images were used to quantify plant biomass, leaf area and health (Rajendran et al. [2009]; Harris et al. [2010]; Golzarian et al. [2011]). The measurement of senescent leaf area in combination with the measurement of shoot Na^+^ concentration enabled the quantification of shoot tissue tolerance in salt stressed einkorn wheat (*Triticum monococcum*) (Rajendran et al. [2009]). Infrared thermography has also been used to measure leaf temperature, as a surrogate for stomatal conductance, to screen the osmotic tolerance of barley and durum wheat seedlings (Sirault et al. [2009]) and rice (Siddiqui et al. [2014]). In the current study, high-throughput image acquisition and analysis was used to study the salinity tolerance traits of two rice cultivars (IR64 and Fatmawati) under different levels of salt stress. The use of this technology for screening individual salt tolerance traits in rice, as well as whole plant salt tolerance, is demonstrated here. These methods can now be used in genetic studies to inform breeding programs of approaches to improve the salinity tolerance of rice.

## Results and discussion

### Imaging as a surrogate for rice shoot biomass measurements

Non-destructive imaging of plants allows multiple measurements of plant growth and plant health on the same individual over time, without having to harvest plant material for analysis (Rajendran et al. [2009]; Furbank and Tester [2011]; Berger et al. [2012b]; Fiorani and Schurr [2013]). Non-destructive imaging is important when measuring the dynamic response of individual plants to the onset of an environmental stress such as salinity and water deficit. It is also important to use non-destructive techniques when plants are unique, such as early generation transgenics, which need to be phenotyped but also maintained for seed collection. Key to the success of this technique is that the measurements obtained are quantitative, quick to obtain and a good surrogate for important traits, such as determination of plant biomass.

A number of studies have used projected shoot area to estimate the shoot biomass of different crops, such as wheat and barley under conditions of salinity stress (Rajendran et al. [2009]; Harris et al. [2010]; Golzarian et al. [2011]).

**Figure 1:**
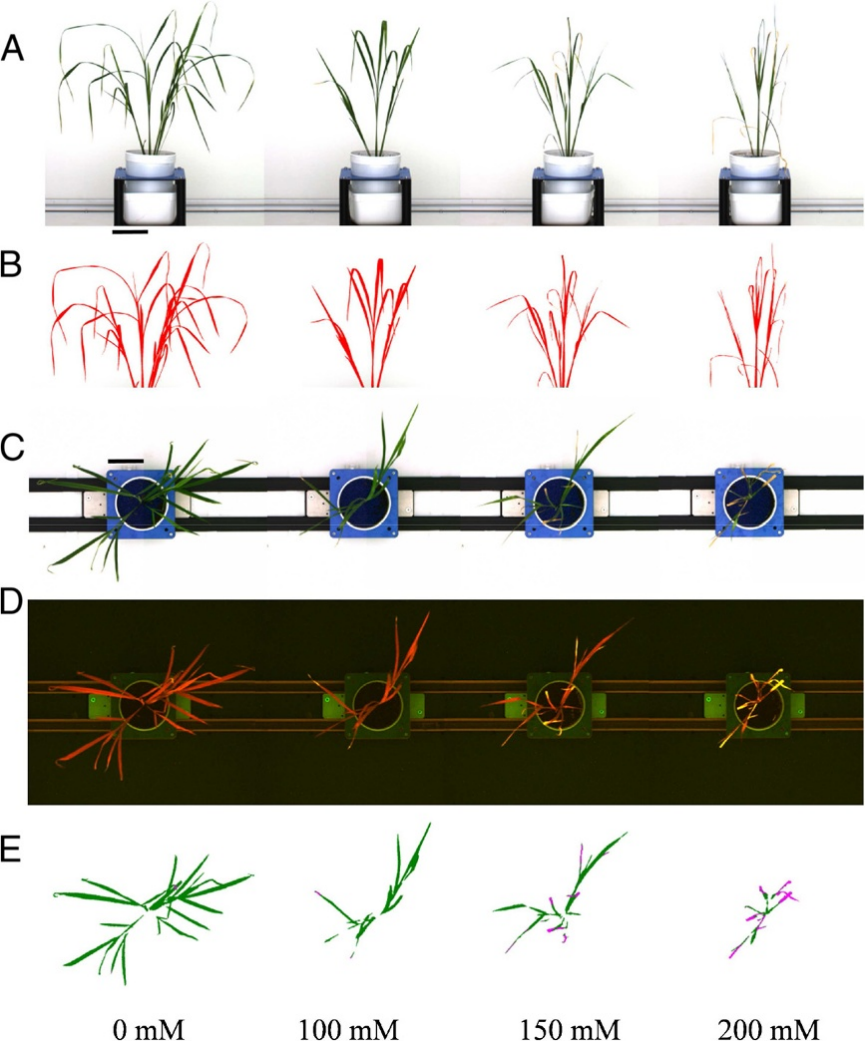
**Example images of rice cv. Fatmawati taken 20 d after salt application.** Salt stress was imposed two weeks after transplantation. From left to right: image of rice treated with 0 mM, 100 mM, 150 mM and 200 mM NaCl. **(A)** Side view RGB images of rice plants. **(B)** Identified object after image analysis for size measurement. **(C)** Top view RGB images of the same plants shown in A. **(D)** Corresponding fluorescent images taken from above. **(E)** Colour classified images derived from fluorescence images in D, where green depicts healthy leaves and purple indicates senescent areas. Bars = 10 cm.

**Figure 2:**
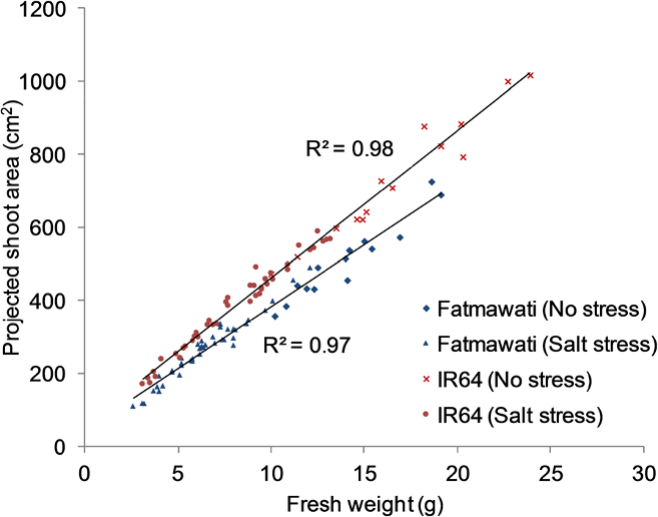
**Relationship between projected shoot areas and shoot fresh weight of rice.** A positive linear relationship was observed in both rice cv. Fatmawati (R^2^ = 0.97, n = 56) and IR64 (R^2^ = 0.98, n = 56). The projected shoot area of rice cv. Fatmawati (blue) and IR64 (red) growing under moderate and high salt stress levels were obtained 20 d after salt application prior to harvest for total shoot fresh weight.

To determine whether shoot biomass of rice plants correlated with the measurements of projected shoot area, and is therefore a quantifiable parameter that can be used to measure plant biomass, RGB images were obtained of plants that had been exposed to various salt stress levels for 20 d (Figure 1) before the fresh weight of each plant was determined by destructive harvest. The projected shoot area was calculated based on two side view images (at 90° from each other) and one top view image (Figure 1). There was a strong positive correlation between the projected shoot area obtained by image analysis and shoot fresh weight in the two rice cultivars, Fatmawati (R^2^ = 0.97) and IR64 (R^2^ = 0.98) (Figure 2) and there was no indication of any deviation from a linear relationship even at the highest biomasses measured in this experiment. Projected shoot area is therefore a suitable surrogate for rice shoot biomass up to six weeks of age and 24 g of shoot fresh weight (Figure 2). It is possible that the correlation between biomass and projected shoot area will still be strong for older plants, however, if running experiments on older plants then this should be confirmed, in case there are issues of areas of the plant being hidden from the camera by other parts of the plant.

### IR64 and Fatmawati differ in their response to salt stress

IR64 and Fatmawati were grown in soil under moderate (0, 50, 75 and 100 mM NaCl) and high (0, 100, 150 and 200 mM NaCl) salt stress. Digital images were taken through time, at 0, 10, and 20 d after salt application.

Measurements of plant growth are important to permit an understanding of the physiological mechanisms underlying the plant response to salt stress over time (Munns [2010]), particularly to separate the effects of the osmotic and ionic stress components, which dominate plant growth at different times (Munns and Tester [2008]).

**Figure 3:**
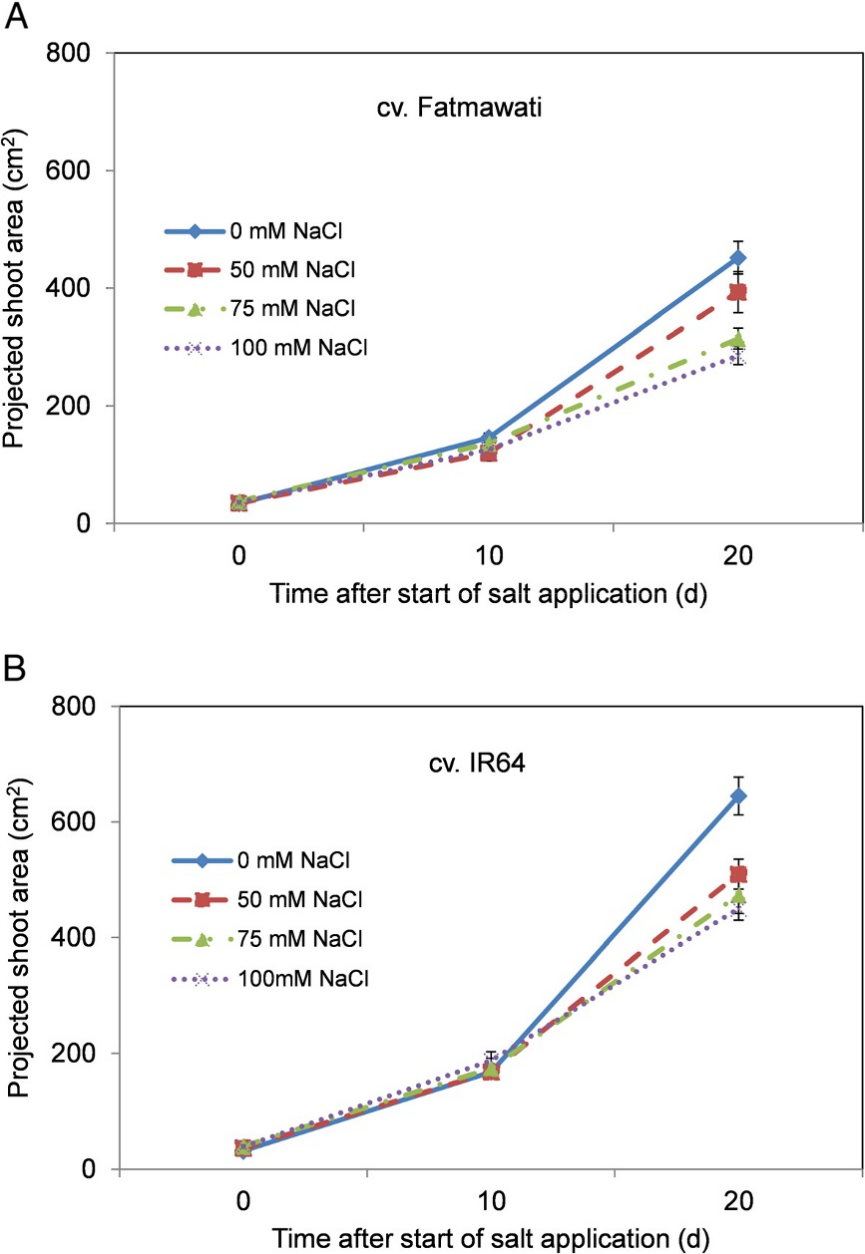
**Projected shoot area of rice cv. (A) Fatmawati and (B) IR64 in moderate salt stress.** The salt levels were 0 mM, 50 mM, 75 mM and 100 mM NaCl, imposed two weeks after transplantation. Values are the means ± SEM (n = 7).

**Figure 4:**
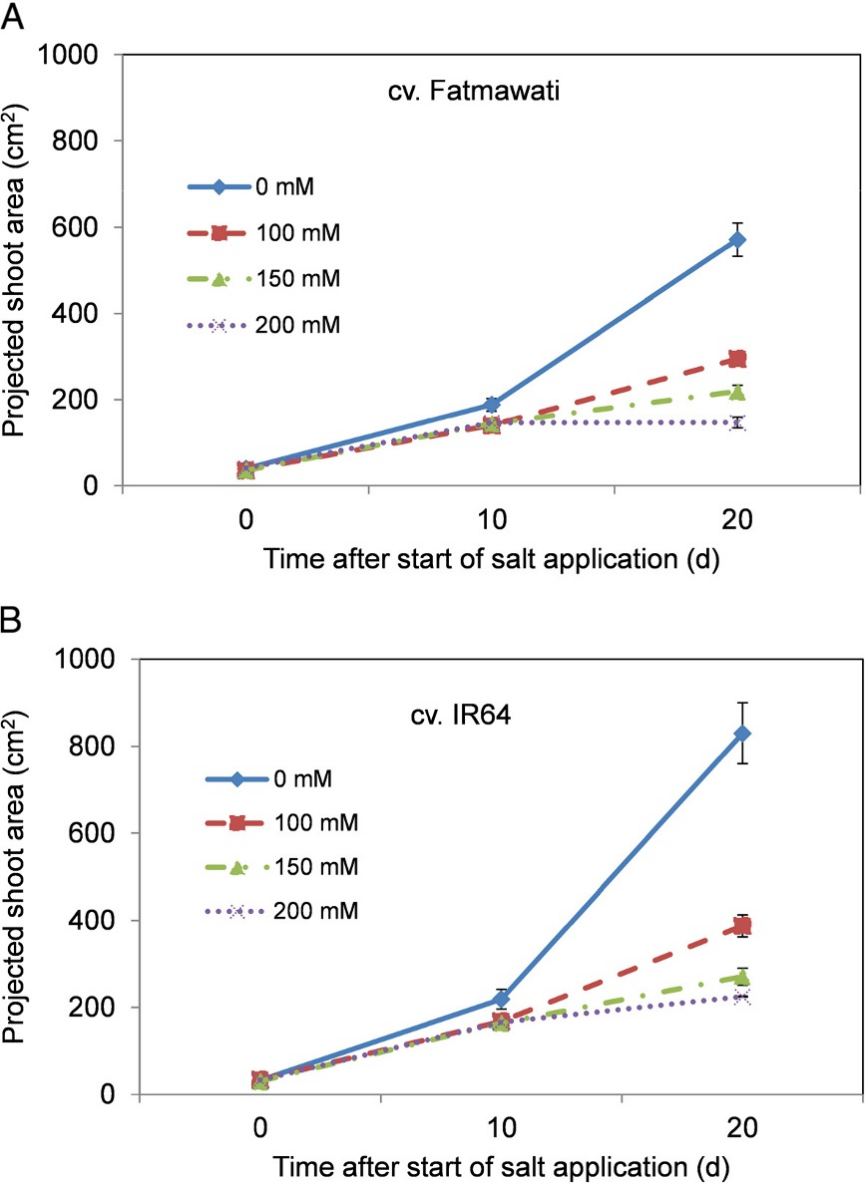
**Projected shoot area of rice cv. (A) Fatmawati and (B) IR64 in high salt stress.** The salt levels were 0 mM, 100 mM, 150 mM and 200 mM NaCl, imposed two weeks after transplantation. Values are the means ± SEM (n = 7).

Interestingly, under these growth conditions there is no apparent reduction in biomass production immediately after salt application (between days 0 and 10), suggesting little response to the osmotic stress component of salt stress in the growing rice plants (Figures 3 and 4). This finding differs to the responses observed in other cereal species, such as einkorn wheat (*T. monococcum*) (Rajendran et al. [2009]), durum wheat (James et al. [2008]; Rahnama et al. [2010]) and bread wheat (Rahnama et al. [2011]), where an immediate response to salt application can be seen - the shoot ion independent stress (osmotic stress) (Munns and Tester [2008]). This early reduction in plant growth is not observed in either IR64 or Fatmawati, suggesting these cultivars have good levels of osmotic tolerance, and that later reductions in growth are likely to be due to ionic stress leading to an increase in premature senescence. A similar observation was reported by Moradi and Ismail ([2007]), where a significant reduction in growth in the seedling stage of rice only became visible after 2 weeks of salt stress. The authors hypothesised that salt tolerant rice plants have the ability to control their stomatal conductance during the initial stress and recover their growth immediately through an acclimation process (Moradi and Ismail [2007]). It is well known that IR64 is a moderately salt tolerant rice variety and has been used intensively in salinity tolerance studies (Asch et al. [2000]; Ueda et al. [2006]; Castillo et al. [2007]; Nakhoda et al. [2012]), however, no information is available on the salinity tolerance of Fatmawati.

After 20 d of salt treatment, however, the projected shoot area of salt stressed rice was clearly reduced compared with that of the non-stressed plants, even at 50 mM NaCl. The reduction in shoot area was more pronounced under the higher concentrations of salt with the shoot area of Fatmawati and IR64 reduced by 37% and 30%, respectively, after 20 d of growth in 100 mM NaCl when compared with non-stressed plants (Figure 3). There was a slight difference in response of rice plants to 100 mM NaCl treatment between two subsequent experiments, which might be attributed to soil batches of different age being used (Figures 3 and 4). In the second experiment (high salt stress; Figure 4), after 20 d of growth under 100 mM NaCl the shoot area of Fatmawati and IR64 was reduced by 49% and 54% compared to control plants, respectively (Figure 4). However, the 0 mM NaCl plants in the second experiment (Figure 4) grew bigger than in the first experiment (low salt stress; Figure 3) for both Fatmawati and IR64, while the plants grown in 100 mM NaCl reached a similar size in both experiments. The increase in biomass reduction is therefore primarily a result of the control plants growing bigger in the second experiment. The late reduction in growth rate in response to salinity in both experiments suggests that the plants experience ionic stress, which can be determined by measurements of leaf senescence and leaf ion concentration.

### Fatmawati exhibits greater shoot senescence under salinity stress than IR64

**Figure 5:**
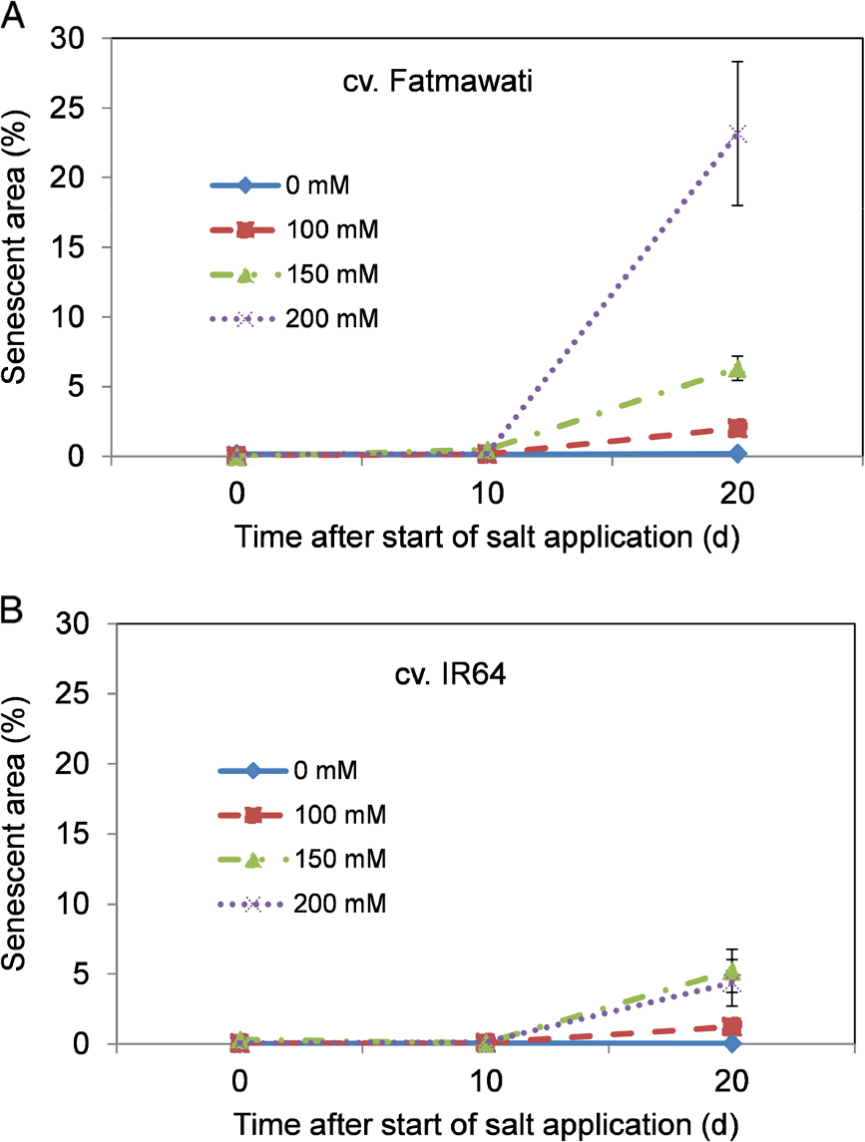
**Percentage of senescent area of rice cv. (A) Fatmawati and (B) IR64 in high salt stress.** The salt levels were 0 mM, 100 mM, 150 mM and 200 mM NaCl, imposed two weeks after transplantation. Senescent area was determined through colour classification from the top view fluorescent images. The results are presented as the percentage of senescent pixels area to the total shoot area of the top view image.

A common method for determining rates of senescence and leaf injury is the visual scoring of symptoms, classifying individuals on an integer scale from zero to an arbitrary upper value (Gregorio et al. [1997]; Negrão et al. [2011]). However, this process is subjective, and can vary depending on the person making the observations. In this study, fluorescence images were used to objectively quantify the degree of leaf senescence of salt stressed plants over time. No difference in senescent area could be observed between stressed and control plants 10 d after salt application. However, the total senescent area was found to increase dramatically by 20 d (Figure 5).

The cultivar Fatmawati appears to be significantly more salt sensitive than IR64, showing considerable shoot senescence (23%) when exposed to 200 mM NaCl for 20 d (Figure 5A). IR64, in contrast, exhibited little shoot senescence (4%), even under very high NaCl concentrations (Figure 5B). While there was an increase in shoot senescent area that corresponded to increasing salt concentrations, both cultivars had little senescence at moderate salinity levels (Figure 5A and 5B).

### IR64 plants accumulate sodium in the shoot while exhibiting low levels of shoot senescence

The concentrations of Na^+^ and K^+^ in the youngest, fully expanded leaf of both IR64 and Fatmawati were measured after 20 d of salt application. Leaf Na^+^ concentrations of the stressed plants increased as expected with increasing external NaCl concentration (Tables 1 and 2). The amount of Na^+^ in the leaf was cultivar dependent, with IR64 accumulating more leaf Na^+^ than Fatmawati (Tables 1 and 2). However, despite accumulating lower concentrations of Na^+^ in the leaf, Fatmawati was found to have higher levels of shoot senescence (Table 2 and Figure 5).This suggests that IR64 may have more efficient Na^+^ tissue tolerance mechanisms, such as the ability to accumulate Na^+^ in the vacuoles of leaf cells (Garbarino and DuPont [1989]; Blumwald et al. [2000]). Sub-cellular measurements of ion accumulation and whole plant/tissue ion fluxes will help elucidate the mechanisms involved (James et al. [2006]; Møller et al. [2009]; Plett et al. [2010]). Interestingly, the concentrations of shoot K^+^ in the stressed plants were higher than those of the control plants, perhaps suggesting that rice attempts to maintain as high a K^+^ concentration as possible during salt stress. However, shoot K^+^/Na^+^ ratios in the stressed plants were significantly lower than those of the non-stressed plants (Tables 1 and 2).

**Table 1:** **Shoot Na** ^**+**^ **and K** ^**+**^ **accumulation and K** ^**+**^ **/Na** ^**+**^ **ratio of rice cv. Fatmawati and IR64 at 20 d after application of a moderate salt stress (50, 75 and 100 mM NaCl)**

Cultivar	Salt stress (mM NaCl)	Na^+^(mM)	K^+^(mM)	Shoot K^+^/Na^+^
Fatmawati	0	1.6 ± 0.1 a	297.4 ± 6.6 a	196.8 ± 16.6
	50	2.4 ± 0.3 b	343.0 ± 6.6 b	151.0 ± 14.1
	75	3.3 ± 0.2 c	362.0 ± 5.6 bc	111.0 ± 5.4
	100	3.4 ± 0.2 c	365.6 ± 7.2 c	111.8 ± 6.6
IR64	0	2.5 ± 0.1 b	345.1 ± 7.4 b	137.4 ± 5.1
	50	4.8 ± 0.3 d	357.3 ± 10.0 bc	76.7 ± 4.4
	75	5.7 ± 0.4 e	365.4 ± 3.5 c	66.4 ± 4.7
	100	6.1 ± 0.4 e	385.5 ± 7.2 d	65.0 ± 4.3
F-test	Cultivar (C)	**	**	**
	Salt stress (S)	**	**	**
	C x S	**	*	ns

Values are the means ± SE (n = 7). Values in the same column followed by the same letter are not significantly different according to LSD (0.05) test. **and * = significant at *P* < 0.01 and 0.05, respectively. ns = not significant.

**Table 2:** **Shoot Na** ^**+**^ **and K** ^**+**^ **accumulation and K** ^**+**^ **/Na** ^**+**^ **ratio of rice cv. Fatmawati and IR64 at 20 d after application of a high salt stress (100, 150 and 200 mM NaCl)**

Cultivar	Salt stress (mM NaCl)	Na^+^(mM)	K^+^(mM)	Shoot K^+^/Na^+^
Fatmawati	0	1.3 ± 0.1	265.3 ± 5.5	208.3 ± 12.3 b
	100	3.8 ± 0.1	326.1 ± 8.2	85.8 ± 2.1 c
	150	8.2 ± 1.0	346.3 ± 9.8	46.3 ± 6.1 d
	200	NA	NA	NA
IR64	0	0.6 ± 0.1	284.4 ± 4.8	465.2 ± 48.0 a
	100	5.9 ± 0.4	320.0 ± 7.0	55.4 ± 4.8 cd
	150	11.6 ± 1.1	325.7 ± 6.0	29.1 ± 2.5 d
	200	12.5 ± 1.3	366.4 ± 11.0	31.2 ± 2.9 d
F-test	Cultivar (C)	**	**	ns
	Salt stress (S)	**	**	**
	C x S	ns	ns	**

Values are the means ± SE (n = 7). NA = data not available due to the high level of senescence in this cultivar. Values in the same column followed by the same letter are not significantly different according to LSD (0.05) test. ** = significant at *P* < 0.01. ns = not significant.

It was not possible to measure Na^+^ and K^+^ concentrations in the last fully expanded leaf of Fatmawati plants grown in 200 mM NaCl due to the high level of senescence (Tables 2 and Figure 5).

### A role for non-destructive phenotyping in screening for rice salinity tolerance

Automation of the phenotyping process in combination with automated plant handling and watering allows large numbers of plants to be screened efficiently with limited handling. Entire populations of plants can be grown in soil media, emulating field conditions (at least for the earlier stages of growth), thus facilitating the transfer of knowledge from controlled environment to growth conditions in the field. An increasing number of phenotyping facilities are now accessible globally, such as the Australian Plant Phenomics Facility (http://www.plantphenomics.org.au), used in this study, or the centres of the European Plant Phenomics Network (http://www.plant-phenotyping-network.eu). The process described here has the potential to be scaled to phenotype large numbers of rice breeding lines and mapping populations allowing the evaluation of the effect of different salt tolerant mechanisms on plant growth and yield. Screening of hundreds of mapping lines and/or rice accessions for bi-parental or association mapping studies can now be done relatively quickly for traits that require time course measurements of growth. The use of such populations has the potential to identify the underlying genetic mechanisms of salinity tolerance in a forward genetics screen.

The main limitation for the widespread adoption of this approach is the cost, but this is decreasing very rapidly as imaging technologies decrease in price and, as knowledge of phenotyping improves, short-cuts and pragmatic compromises can be more confidently undertaken. This will be accompanied by an increasing ability to phenotype cheaply in the field – although this inevitably comes with a reduced ability to control and manipulate environmental conditions. As costs decrease, so the power of this approach will also increase, to enable more detailed physiological characterization of rice genotypes (e.g. stomatal behaviour) in response to salinity (e.g. by combination of infrared (IR), RGB and fluorescence techniques).

## Conclusions

An efficient and high-throughput screening protocol to select salt-tolerant rice is required to accelerate the development of salt-tolerant rice cultivars. A non-destructive image-based phenotyping method to analyse the responses of rice to different levels of salinity stress has been developed and revealed differences in the effects of salt stress in two cultivars of rice, IR64 and Fatmawati. Use of non-destructive imaging technologies, such as those described here, in combination with measurements of tissue ion concentration, allow the differentiation between the ionic and osmotic components of salt stress in growing rice. This will enable the identification of new traits and sources of salinity tolerance genes that can be used to pyramid different salinity tolerance mechanisms into elite rice breeding lines.

## Methods

### Plant material and growth conditions

Two Indica rice cultivars, IR64 and Fatmawati, were used in this study. IR64 was developed at the International Rice Research Institute (IRRI) in the Philippines and is known as a “mega variety” because of its wide adoption and cultivation in large areas (Mackill [2008]). Fatmawati is a new plant type of rice from Indonesia (Abdullah et al. [2008]) which has new morphological traits, such as fewer tillers, sturdy culms and higher number of seed per panicle compared to traditional rice cultivars (Khush [1995]; Abdullah et al. [2008]). IR64 and Fatmawati seeds were obtained from the Indonesian Centre for Rice Research, Sukamandi, Indonesia.

Rice seeds were sorted for uniform size and then dehusked and surface sterilised with 70% (v/v) ethanol for one min followed by a 30 min bleach treatment (30% (v/v) commercial Domestos bleach solution, sodium hypochlorite 49.9 g/L). The seeds were then washed with RO water five times to remove all traces of bleach. Surface sterilised seeds were germinated by placing them on sterile wet filter paper in dishes. The dishes were sealed to prevent evaporation and contamination and placed in a growth chamber with 12 h light and a constant temperature of 28°C. After seven days, uniformly germinated seedlings were transferred into white plastic pots (150 mm diameter × 200 mm height) containing 3 kg dry University of California (U.C.) soil mix (Matkin and Chandler [1957]), composed of sand and peat moss (volume ratio 1.6:1) and fertiliser (1.5 kg Mini Osmocote® per 600 litre UC soil base). The soil surface was covered by blue plastic pellets to reduce development of algae, reduce evaporation and to provide a favourable background colour for image analysis. Three rice seedlings were transplanted into each pot but were thinned to a single plant per pot after one week.

The plastic pots had drainage holes in their base, allowing watering from underneath (Figure 1A). The pots were placed in a deep white plastic saucer (160 mm × 160 mm × 90 mm) to which a volume of 600 mL of water was applied to each pot via the saucer. Water levels were monitored daily by weighing the pots using a digital scale, and pots were adjusted to the target weight by adding water to maintain a constant salt concentration in each pot.

The rice plants were placed in a growth chamber (Conviron, Model PGC20) at The Plant Accelerator® (Australian Plant Phenomics Facility, University of Adelaide, Adelaide, Australia) with a sinusoidal 12 hr/12 hr day/night cycle. Growth chambers were fitted with Osram FQ54W/840 HO fluorescence lights (Osram Australia, Pennant Hills, Australia) providing an average photon irradiance of 200 - 300 μmol/m^2^/s over a 24 h period. Humidity was maintained at 70% with day/night temperatures of 28°C/26°C, respectively.

### Salt treatment

Two separate salt experiments (moderate salt stress and high salt stress) were carried out sequentially in 2012. The moderate salt experiment was conducted from 15 February to 26 March 2012 and the high salt experiment was from 23 March to 3 May 2012. In the moderate salt stress experiment, the rice cultivars were subjected to four levels of salt, 0 mM, 50 mM, 75 mM and 100 mM NaCl. In the high salt stress experiment the salt levels were 0 mM, 100 mM, 150 mM and 200 mM NaCl. Seven biological replicates were used for each treatment. Salt treatments were imposed 14 d after seedling transplantation in 50 mM increments every 12 h until the desired level was reached to minimise the osmotic shock to the plants. For each increment, 60 mL of a 0.5 M NaCl solution was applied to the saucer in which the pots sat. Additional CaCl_2_ was added to prevent Na^+^ induced Ca^2+^deficiency with the ratio of Na^+^:Ca^2+^ molar concentration of 30:1 (3.3 mM CaCl_2_ for 100 mM NaCl (Genc et al. [2010])). The concentrations of NaCl in the soil were maintained at constant levels by watering each pot to weight, as described in the previous section.

### Image capture and image analysis

Shoot images were taken using the LemnaTec 3D Scanalyzer system (LemnaTec GmbH, Aachen, Germany) at The Plant Accelerator® at 14 d after seedling transplantation (before the start of salt application), 10 d and 20 d after the start of salt application. The pots were manually loaded onto the conveyer belt and automatically moved to the image capture stations. Three 5 mega pixel colour images (RGB images) were taken per plant, two from the side at 90° from each other and one from the top. Three fluorescent images were taken in a separate imaging chamber with constant blue light excitation (400 nm to 500 nm) and a 1.4 mega pixel colour camera with a 500 nm high pass filter capturing steady-state fluorescence emission from 500 nm to 750 nm of light-adapted plants. The images captured allow detection of senescence, necrosis and chlorosis but are not suitable for measuring photosynthetic activity since the lighting system is not pulsed. After image capture, all images were analysed using the LemnaTec Grid software package (LemnaTec GmbH, Aachen, Germany).

In brief, the plant was separated from the imaging background using a nearest-neighbour colour classification. Noise was removed from the images using erosion and dilation steps as well as a size filter. Subsequently, all objects identified as being part of the plant were composed to one single object. The visible RGB images were used to measure size and height of the object (Berger et al. [2012a]). The summed area of all three images per plant was used as an approximation for shoot biomass (Rajendran et al. [2009]; Golzarian et al. [2011]; Berger et al. [2012a]). The top view fluorescent images were used to quantify the level of shoot senescence. After object separation from the background and noise reduction, nearest-neighbour colour classification was used to separate the shoot into healthy leaf area (red chlorophyll fluorescence) and senescent leaf area (yellow fluorescence; Figure 1D). The level of senescence was calculated as the percentage of senescence pixels relative to total shoot area.

### Measurement of shoot biomass and shoot ion concentration

Shoots were harvested 20 d after salt application and the fresh weight was measured using a digital scale. Leaf tissue for shoot Na^+^ and K^+^ measurements were taken from the youngest fully expanded leaf at 20 d after salt application. The leaves were weighed immediately after harvest to determine their fresh weight and then dried in an oven at 70°C for 24 h. The dry weight was measured to determine the tissue water content. Dried leaf samples were placed in 50 mL Falcon tubes and digested in 20 mL1% (v/v) nitric acid (HNO_3_) for 5 h in a heat block at 70°C. The samples were shaken every hour to ensure complete digestion. The concentrations of Na^+^ and K^+^ were determined using a flame photometer (model 420; Sherwood Scientific Ltd., Cambridge, UK).

## Authors’ original submitted files for images

Below are the links to the authors’ original submitted files for images. Authors’ original file for figure 1 Authors’ original file for figure 2 Authors’ original file for figure 3 Authors’ original file for figure 4 Authors’ original file for figure 5
