# Supplementary figures and images for: Image-based phenotyping for non-destructive screening of different salinity tolerance traits in rice
Source: Rice (N Y). 2014 Aug 14;7:16. doi: 10.1186/s12284-014-0016-3 (PMC4884049; doi:10.1186/s12284-014-0016-3)

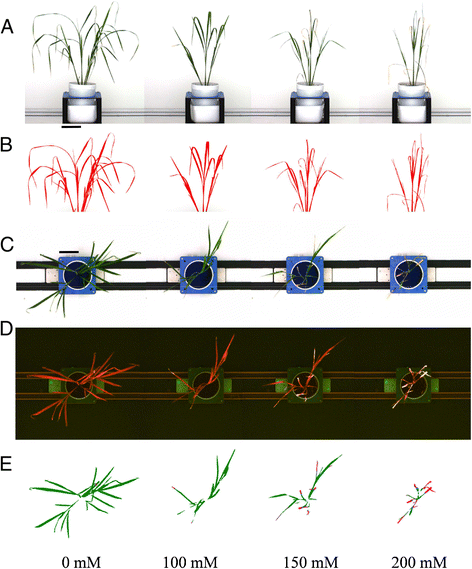

Supplement: Supplementary file 1 — Authors’ original file for figure 1 [file 12284_2014_16_MOESM1_ESM.gif]

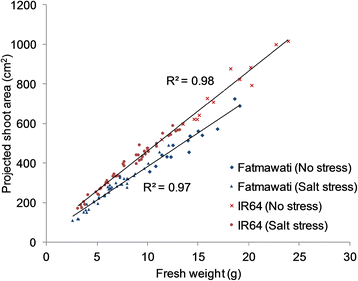

Supplement: Supplementary file 2 — Authors’ original file for figure 2 [file 12284_2014_16_MOESM2_ESM.gif]

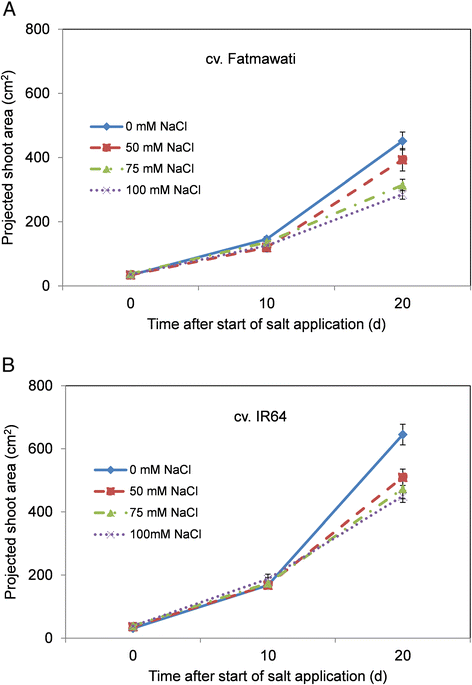

Supplement: Supplementary file 3 — Authors’ original file for figure 3 [file 12284_2014_16_MOESM3_ESM.gif]

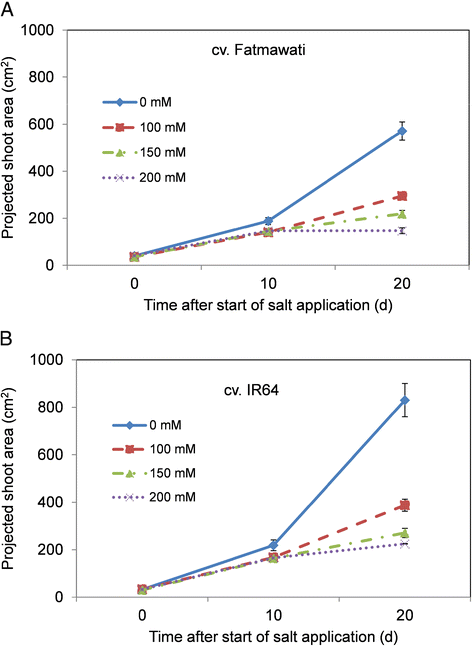

Supplement: Supplementary file 4 — Authors’ original file for figure 4 [file 12284_2014_16_MOESM4_ESM.gif]

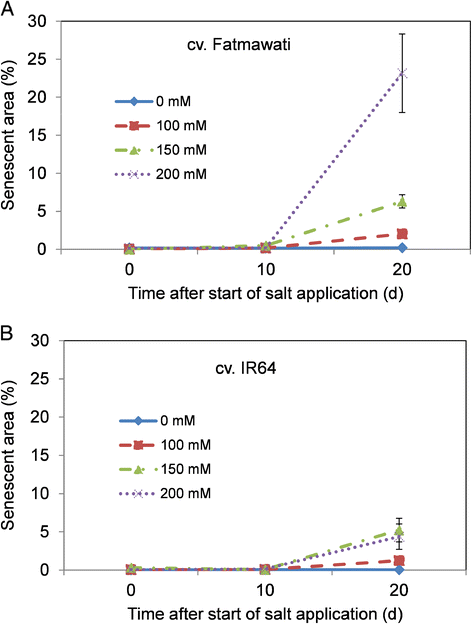

Supplement: Supplementary file 5 — Authors’ original file for figure 5 [file 12284_2014_16_MOESM5_ESM.gif]
